# Functional Properties of CD8^+^ Lymphocytes in Patients with Pleural Plaque and Malignant Mesothelioma

**DOI:** 10.1155/2014/670140

**Published:** 2014-06-18

**Authors:** Naoko Kumagai-Takei, Yasumitsu Nishimura, Megumi Maeda, Hiroaki Hayashi, Hidenori Matsuzaki, Suni Lee, Takumi Kishimoto, Kazuya Fukuoka, Takashi Nakano, Takemi Otsuki

**Affiliations:** ^1^Department of Hygiene, Kawasaki Medical School, Kurashiki 701-0192, Japan; ^2^Laboratory of Functional Glycobiochemistry, Division of Agricultural and Life Science, Department of Biofunctional Chemistry, Graduate School of Environmental and Life Science, Okayama University, Okayama 700-8530, Japan; ^3^Department of Dermatology, Kawasaki Medical School, Kurashiki 701-0192, Japan; ^4^Okayama Rosai Hospital, Okayama 702-8055, Japan; ^5^Department of Respiratory Medicine, Hyogo College of Medicine, Nishimomiya 663-8501, Japan

## Abstract

It is known that asbestos exposure can cause malignant mesothelioma (MM) and that CD8^+^ T cells play a critical role in antitumor immunity. We examined the properties of peripheral blood CD8^+^ lymphocytes from asbestos-exposed patients with pleural plaque (PL) and MM. The percentage of CD3^+^CD8^+^ cells in PBMCs did not differ among the three groups, although the total numbers of PBMCs of the PL and MM groups were lower than those of the healthy volunteers (HV). The percentage of IFN-*γ* ^+^ and CD107a^+^ cells in PMA/ionomycin-stimulated CD8^+^ lymphocytes did not differ among the three groups. Percentages of perforin^+^ cells and CD45RA^−^ cells in fresh CD8^+^ lymphocytes of PL and MM groups were higher than those of HV. Percentages of granzyme B^+^ and perforin^+^ cells in PMA/ionomycin-stimulated CD8^+^ lymphocytes were higher in PL group compared with HV. The MM group showed a decrease of perforin level in CD8^+^ lymphocytes after stimulation compared with patients with PL. These results indicate that MM patients have characteristics of impairment in stimulation-induced cytotoxicity of peripheral blood CD8^+^ lymphocytes and that PL and MM patients have a common character of functional alteration in those lymphocytes, namely, an increase in memory cells, possibly related to exposure to asbestos.

## 1. Introduction

Asbestos fibers exhibit tumorigenicity. It is commonly believed that the cause of lung cancer and malignant mesothelioma induced by inhaled asbestos might be due to its tumorigenic activity [1, 2]. Previous reports have shown that asbestos induces oxidization of nucleotide bases and increases mutation frequencies [3, 4]. However, it takes a long period of about forty years to develop malignant mesothelioma after exposure to asbestos [5–7]. These findings suggest that asbestos might gradually impair antitumor immunity [8–14].

In antitumor immunity, cytotoxic T lymphocytes (CTL) and natural killer (NK) cells play a role as effectors, which kill tumor cells [15]. CTL are known to be differentiated from naïve CD8^+^ T cells. When these naïve cells recognize antigen, they are immediately activated, expand, and differentiate into antigen-specific CTL [16]. Following conjugation of CTL with an appropriate target cell, lytic granules are transported to the point of contact with the target cell, and the granule contents, perforin, and granzyme B are released into the immune synapse between CTL and the target. Once CTL release granule contents, they act on target cells and induce apoptosis to eliminate these cells [17]. The granule core is surrounded by a lipid bilayer containing lysosome-associated membrane glycoprotein, CD107a, which is known to be transiently expressed on the T cell surface upon degranulation [18].

We reported recently that asbestos exposure suppressed the differentiation of human CTL during mixed lymphocyte reactions (MLR), which was accompanied by decreases in IFN-*γ* and tumor necrosis factor-*α* [19]. Several additional findings include low cytotoxicity and altered expression of NK cell-activating receptors on NK cells and a decrease in CD4^+^ CXCR3^+^ T cells in patients with mesothelioma [8, 9]. These findings raise the possibility that CD8^+^ lymphocytes might show a functional decline in asbestos-exposed patients with pleural plaque (PL) or malignant mesothelioma (MM). Pleural plaques are an objective sign of previous asbestos inhalation, are known to be whitish, sharply circumscribed, fibrous, hyaline, sometimes calcified, form patches involving parietal pleura, and there is a report that pleural plaques are harmless in themselves [20].

Therefore, in the present study, we examined the functional properties of CD8^+^ lymphocytes of patients with PL or MM by flow cytometry (FCM) and compared results with those of healthy volunteers (HV). Freshly prepared peripheral blood monocyte cells (PBMCs) were assayed for the percentage and number of CD3^+^CD8^+^ cells and percentages of granzyme B^+^, perforin^+^, and CD45RA^−^ cells in CD8^+^ lymphocytes. PBMCs were also stimulated with phorbol 12-myristate 13-acetate (PMA)/ionomycin for 4 h and assayed for the percentages of IFN-*γ* ^+^, CD107a^+^, granzyme B^+^, and perforin^+^ cells in CD8^+^ lymphocytes.

## 2. Materials and Methods

### 2.1. Isolation of PBMCs

Blood from HV and patients with PL or MM was collected in citrate phosphate dextrose, and PBMCs were isolated from blood using a Ficoll-Hypaque density gradient. Specimens were taken from 16 HV (mean ± S.D. age, 57.2 ± 6.4 years; 16 men), 27 patients with PL (mean ± S.D. age, 66.7 ± 5.1 years; 27 men), and 18 patients with MM (mean age, 67.3 ± 9.0 years; 18 men). Isolated PBMCs were assayed for the percentage and number of CD3^+^CD8^+^ cells and percentages of CD45RA^−^, granzyme B^+^, and perforin^+^ cells in CD8^+^ lymphocytes. A portion of PBMCs were cultured and stimulated as described below and assayed for the percentages of IFN-*γ* ^+^, CD107a^+^, granzyme B^+^, and perforin^+^ cells in CD8^+^ lymphocytes. All donors provided their informed consent and the Institutional Ethics Committees of Kawasaki Medical School, Okayama Rosai Hospital, and Hyogo College of Medicine approved the project.

### 2.2. Cell Culture

For staining of intracellular IFN-*γ*, granzyme B, perforin, and cell-surface CD107a, isolated 2.0 × 10^5^ PBMCs were stimulated with 50 ng/mL PMA and 250 ng/mL ionomycin (both Sigma-Aldrich, St. Louis, MO) in RPMI-1640 medium supplemented with 10% FBS (Molecular and Biological laboratories, Co., Ltd.), 100 *μ*g/mL streptomycin, and 100 U/mL penicillin (both Meiji, Tokyo, Japan) in 96-well flat-bottomed plates. The plates were incubated at 37°C for 4 h in a humidified atmosphere of 5% CO_2_. Golgi Stop Protein Transporter inhibitor (containing monensin) (BD Biosciences, San Jose, CA) was also added to the medium for the staining of IFN-*γ*.

### 2.3. Assay for Expression Levels of Cell-Surface and Intracellular Molecules

Isolated PBMCs were stained with the following antibodies (Abs): CD8-phycoerythrin cychrome 5 (PC5) (Beckman Coulter, Inc., Brea, CA) and CD3-FITC (BD Biosciences), CD45RA-phycoerythrin (PE) (BioLegend, San Diego, CA), granzyme B-RPE (AbD Serotec, Oxford, UK), IgG1 negative control: RPE (AbD Serotec), perforin-RPE (Ancell Corporation, Bayport, MN), or IgG2b-RPE (Ancell Corporation) Abs. The stimulated PBMCs were harvested and stained with CD8-PC5 and IFN-*γ*-PE Abs (BD Biosciences) using a Cytofix/Cytoperm Fixation/Permeabilization kit (BD Biosciences). The stimulated cells were also stained with CD8-PC5 and perforin-RPE or granzyme B-RPE Abs. Before staining with granzyme B-RPE or perforin-RPE Ab, the cells stained with CD8-PC5 Ab were fixed with 3.7% formaldehyde and then permeabilized with 0.1% Triton X-100. The percentage of cells positive for each parameter was analyzed using a FACSCalibur (BD Biosciences).

### 2.4. Detection of Degranulation

The degranulation of CD8^+^T lymphocytes stimulated by PMA and ionomycin was detected by the increase in expression of cell-surface CD107a using the FACSCalibur. Isolated PBMCs were stimulated in the presence of PMA and ionomycin for 4 h, as described above. At the end of the stimulation period, cells were washed and stained with CD107a-PE (BD Biosciences) and CD8-PC5 Abs. Cells positive for CD107a expression were defined by comparison with unstimulated control cells.

### 2.5. Statistical Analysis

The significance of differences was determined using an analysis of variance (ANOVA) with a* post hoc* Student-Newman-Keuls test. The statistical analysis was performed using StatView 5.0 (SAS Institute Inc., Cary, NC) software.

## 3. Results

### 3.1. Percentage of CD3^+^CD8^+^ Cells in PBMCs and the Number of CD3^+^CD8^+^ Cells in Blood

**Figure 1:**
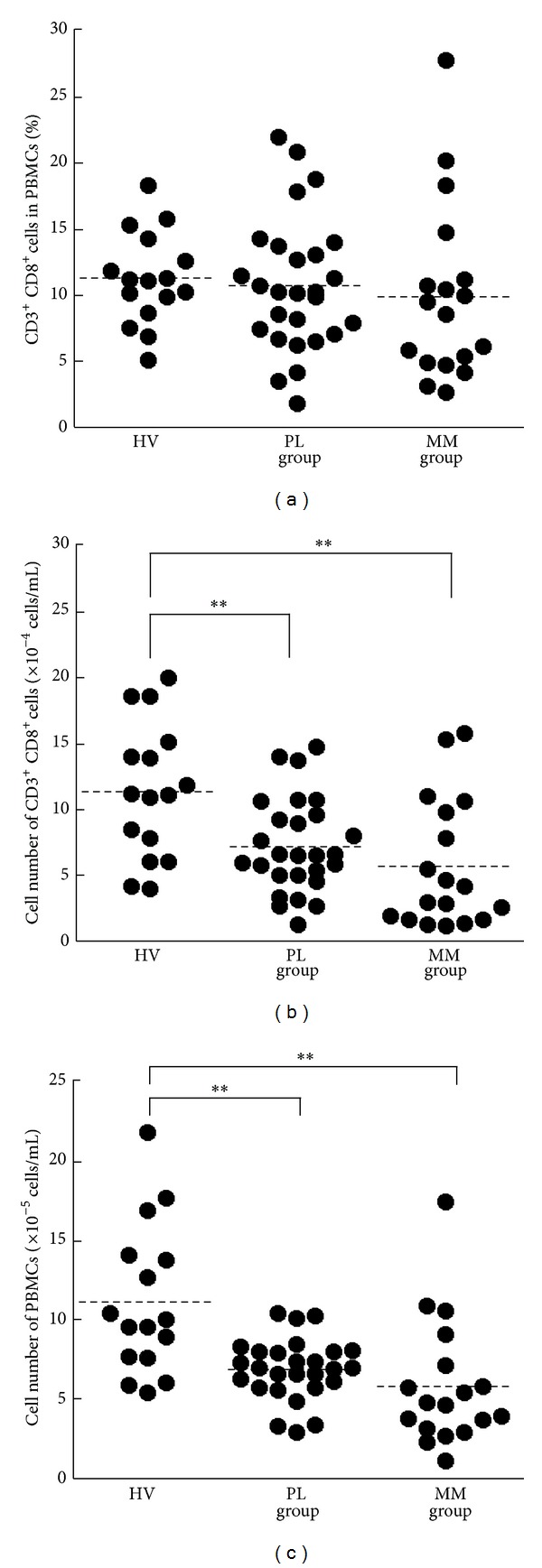
The percentage and number of CD3^+^CD8^+^ cells for HV, PL, and MM groups. Freshly isolated PBMCs were assayed for the percentage of CD3^+^CD8^+^ cells in PBMCs (a) and the number of CD3^+^CD8^+^ cells per 1 mL of blood (b) and the number of PBMCs per 1 mL of blood (c). Horizontal dotted bars indicate the mean percentage (a) and mean number ((b), (c)). Data represent values from 16 HV individuals, 27 individuals of the PL group, and 18 individuals of the MM group. Significant differences are indicated by asterisks ( **P < 0.01).

We isolated fresh PBMCs from the peripheral blood of HV, PL, and MM groups and examined the percentage of CD3^+^CD8^+^ cells in PBMCs and the number of CD3^+^CD8^+^ cells in blood. The percentage of CD3^+^CD8^+^ cells in PBMCs did not differ among HV, PL, and MM groups (mean ± S.D., 11.3 ± 3.5, 10.7 ± 5.1, and 9.9 ± 6.7, resp.) (Figure 1(a)). However, statistical analysis showed a significant difference between groups (*P* < 0.01) for the number of CD3^+^CD8^+^ cells. The number of CD3^+^CD8^+^ cells per 1 mL of blood in PL and MM groups was significantly lower than that of HV (mean ± S.D., 7.2 ± 3.6, 5.6 ± 4.9, and 11.5 ± 4.9, resp.) (Figure 1(b)). These results reflect the decrease in total number of PBMCs, which was significantly lower for the PL and MM groups than the HV group (Figure 1(c)). Thus, the proportion of CD3^+^CD8^+^ cells in PL and MM patients did not differ, although the total number of PBMCs decreased.

### 3.2. CD45RA-Negative Cells in CD8^+^ Lymphocytes

**Figure 2:**
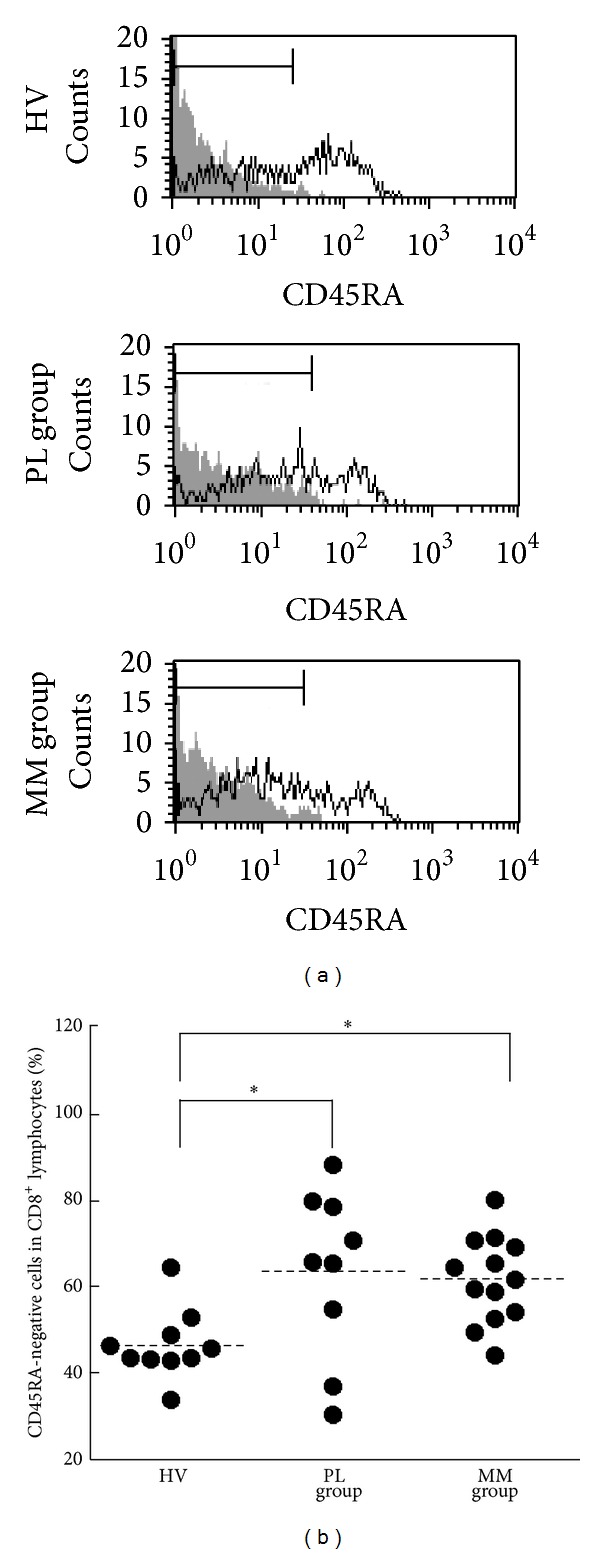
The percentages of CD45RA-negative cells in CD8^+^lymphocytes of HV, PL, and MM groups. Freshly isolated PBMCs were assayed for the percentage of CD45RA-negative cells in CD8^+^ lymphocytes. (a) Representative histograms of cell-surface CD45RA (solid line) and nonstaining control (gray) in CD8^+^ lymphocytes. For the nonstaining control, PBMCs from arbitrary HV samples were used. (b) Representative graph showing the percentage of CD45RA-negative cells in CD8^+^ lymphocytes. Horizontal dotted bars indicate the mean percentage. Data represent values from 10 HV individuals, 9 individuals of the PL group, and 13 individuals of the MM group. Significant differences are indicated by asterisks ( *P < 0.05).

CD45RA-negative cells in CD8^+^ T cells are known as memory cells in peripheral blood [21, 22]. We considered the possibility that the percentages of CD45RA-negative cells in CD8^+^ lymphocytes of asbestos-exposed patients with PL and MM might be lower than those of HV because our previous study using MLR showed that asbestos exposure suppressed the differentiation of human CTL [19]. To examine this possibility, we assayed the percentages of CD45RA-negative cells in CD8^+^ T cells among the three groups (Figure 2(a)). There was a significant difference in percentages of CD45RA-negative cells in fresh CD8 lymphocytes among the groups. Contrary to our expectation, the percentages of CD45RA-negative cells in fresh CD8^+^ lymphocytes of the PL and MM groups were significantly higher than those of HV (mean ± S.D., 63.4 ± 19.5, 61.7 ± 10.0, and 46.5 ± 8.0, resp.) (Figure 2(b)).

### 3.3. Production of IFN-*γ* by CD8^+^ Lymphocytes

**Figure 3:**
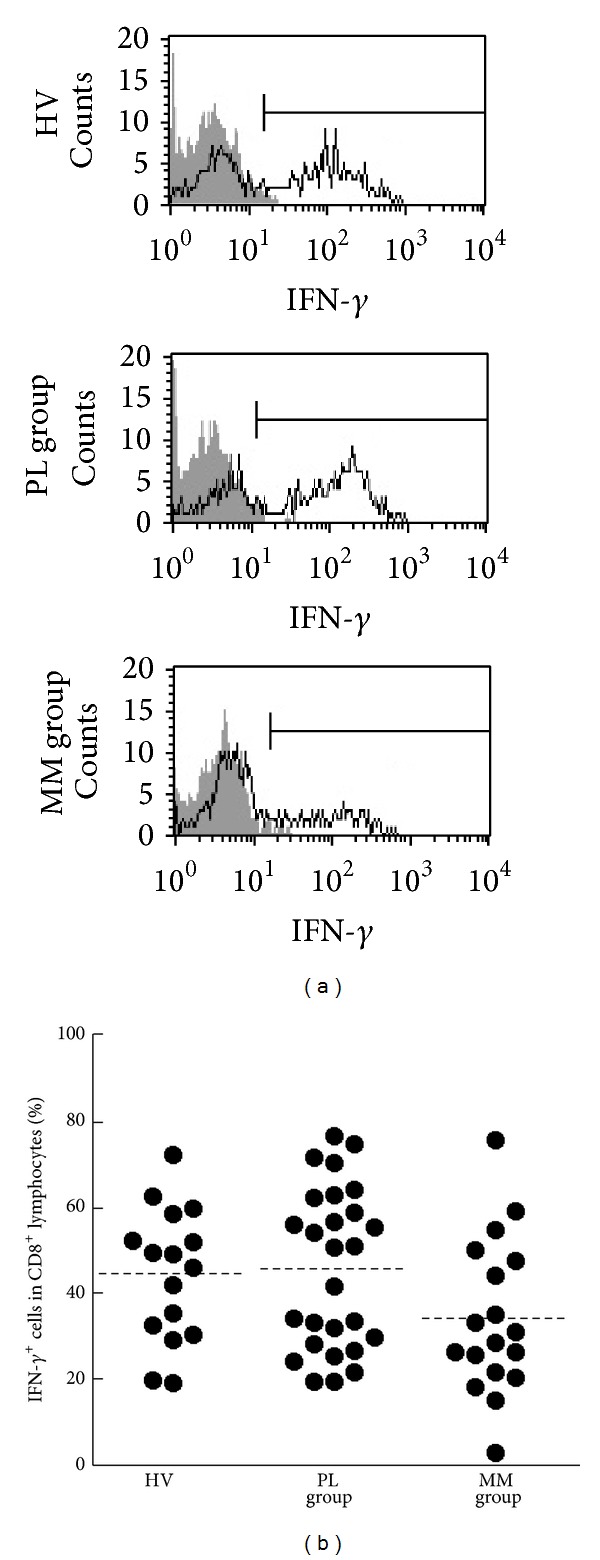
The percentages of IFN-*γ*-positive cells in CD8^+^lymphocytes of HV, PL, and MM groups. (a) Representative histograms of intracellular IFN-*γ* (solid line) and nonstaining control (gray) in CD8^+^ lymphocytes. (b) Representative graph showing the percentage of IFN-*γ* ^+^ cells in CD8^+^ lymphocytes. Horizontal dotted bars indicate the mean percentage. Data represent values from 16 HV individuals, 27 individuals of the PL group, and 18 individuals of the MM group.

CD8^+^ T cells have been shown to be crucial for the immune response against tumors through production of effector molecules including granzyme B, perforin, and IFN-*γ* [23, 24]. To examine the ability of these cells to produce IFN-*γ* in patients with PL and MM, we assayed the percentages of cells positive for intracellular IFN-*γ* in stimulated CD8^+^ lymphocytes of individuals in the PL, MM, and HV groups. After PBMCs were stimulated with PMA/ionomycin as a substitute for antigenic stimulation for 4 h, they were analyzed for the percentage of IFN-*γ*-positive cells in CD8^+^ lymphocytes and the results were compared between the HV, PL, and MM groups (Figure 3(a)). There was no statistically significant difference among the groups, although the percentage of IFN-*γ* ^+^ cells in the MM group showed a tendency to be lower than that of the HV and PL groups (mean ± S.D., 34.3 ± 18.0, 44.5 ± 15.5, and 45.8 ± 18.7, resp.) (Figure 3(b)).

### 3.4. Storage and Retention/Enhancement of Granzyme B by CD8^+^ Lymphocytes

**Figure 4:**
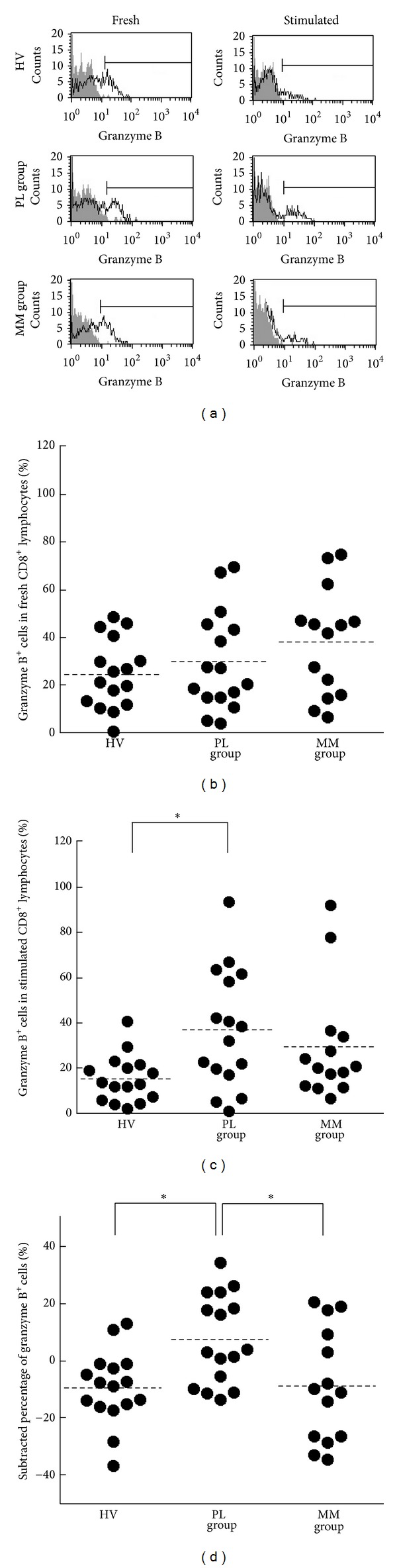
The percentages of granzyme B-positive cells in CD8^+^lymphocytes of HV, PL, and MM groups. (a) Representative histograms of intracellular granzyme B (solid line) and isotype control (gray) in fresh or stimulated CD8^+^ lymphocytes. For staining with an isotype control Ab, PBMCs from arbitrary HV samples were used. (b) Representative graph showing the percentage of granzyme B^+^ cells in fresh CD8^+^ lymphocytes. (c) Representative graph showing the percentage of granzyme B^+^ cells in PMA/ionomycin-stimulated CD8^+^ lymphocytes. (d) Representative graph showing the subtracted percentages of granzyme B^+^ cells. ((b)–(d)) Horizontal dotted bars indicate the mean percentage. Data represent values from 16 HV individuals, 16 individuals of the PL group, and 14 individuals of the MM group. Significant differences are indicated by asterisks ( *P < 0.05).

To examine the storage level of intracellular granzyme B and its retention/enhancement in CD8^+^ lymphocytes of patients with PL and MM, we assayed the percentage of cells positive for intracellular granzyme B in fresh or stimulated CD8^+^ lymphocytes (Figure 4(a)). The percentages of granzyme B^+^ cells in fresh CD8 lymphocytes did not differ among HV, PL, and MM groups (mean ± S.D., 24.6 ± 14.5, 29.6 ± 20.8, and 38.0 ± 22.8, resp.) (Figure 4(b)). In contrast to results for fresh CD8^+^ lymphocytes, there was a significant difference in the percentages of granzyme B^+^ cells in PMA/ionomycin-stimulated CD8 lymphocytes among the groups. The PL group showed a higher percentage of granzyme B^+^ cells in stimulated CD8^+^ cells compared to HV (mean ± S.D., 37.0 ± 26.3, and 15.2 ± 10.4, resp.), although the percentage did not differ from that of the MM group (mean ± S.D., 29.2 ± 25.3) (Figure 4(c)). To examine the retaining/enhancing performance of granzyme B molecules in CD8^+^ lymphocytes after stimulation, the percentage of granzyme B^+^ cells in fresh CD8^+^ lymphocytes was subtracted from the percentage of granzyme B^+^ cells in stimulated cells of each individual (Figure 4(d)). Figure 4(d) reveals a significant difference in the subtracted percentages of granzyme B^+^ cells among the groups and shows that the subtracted percentage of granzyme B^+^ cells of the PL group was significantly higher than that of the HV or MM group. These results indicate that CD8^+^ lymphocytes in PL patients have an increased ability to retain and enhance intracellular granzyme B following stimulation, compared with HV and MM patients.

### 3.5. Storage and Retention/Enhancement of Perforin by CD8^+^ Lymphocytes

**Figure 5:**
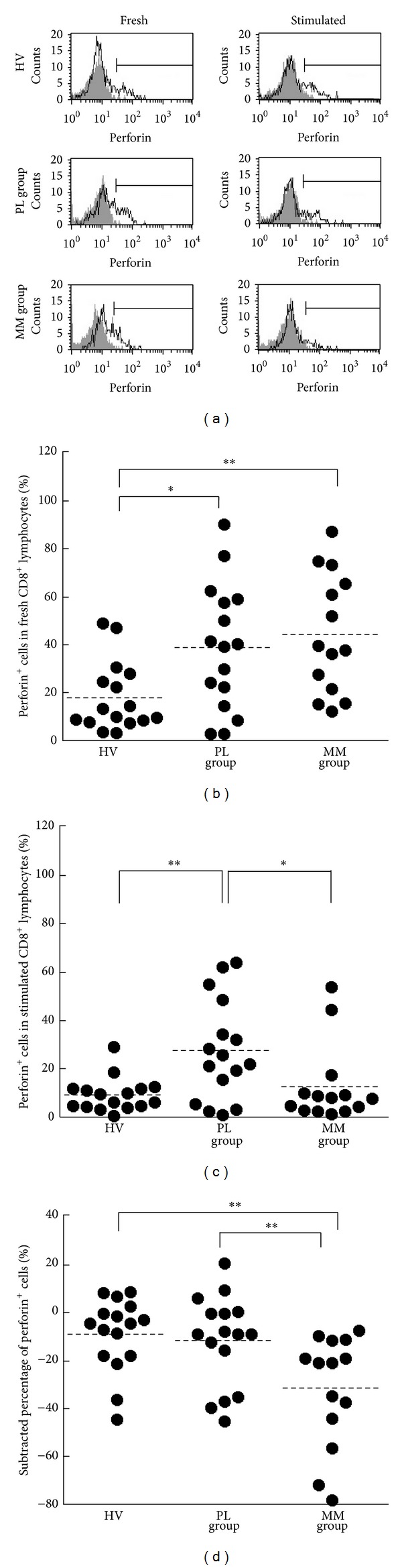
The percentages of perforin-positive cells in CD8^+^lymphocytes of HV, PL, and MM groups. (a) Representative histograms of intracellular perforin (solid line) and isotype control (gray) in fresh or stimulated CD8^+^ lymphocytes. For staining with an isotype control Ab, PBMCs from arbitrary HV samples were used. (b) Representative graph showing the percentage of perforin^+^ cells in fresh CD8^+^ lymphocytes. (c) Representative graph showing the percentage of perforin^+^ cells in PMA/ionomycin-stimulated CD8^+^ lymphocytes. (d) Representative graph showing the subtracted percentages of perforin^+^ cells. ((b)–(d)) Horizontal dotted bars indicate the mean percentage. Data represent values from 16 HV individuals, 16 individuals of the PL group, and 14 individuals of the MM group. Significant differences are indicated by asterisks ( *P < 0.05,  **P < 0.01).

In a manner similar to the analysis of granzyme B level, the percentages of cells positive for intracellular perforin in fresh or stimulated CD8^+^ lymphocytes were assayed, and each percentage and subtracted percentage were compared among the PL, MM, and HV groups (Figure 5(a)). The percentages of perforin^+^ cells in fresh CD8 lymphocytes of the PL and MM groups were significantly higher than those of the HV group (mean ± S.D., 38.8 ± 26.3, 44.2 ± 24.9, and 17.8 ± 14.5, resp.) (Figure 5(b)). In addition, the PL group, but not the MM group, showed a higher percentage of perforin^+^ cells in stimulated CD8^+^ lymphocytes compared to the HV group (mean ± S.D., 27.4 ± 20.9, 12.6 ± 16.2, and 9.1 ± 7.0, resp.) (Figure 5(c)). Moreover, there was a significant difference in the subtracted percentage, calculated by subtraction of the amount in fresh cells from that in stimulated cells, among the groups. The MM group showed significantly lower subtracted percentages of perforin^+^ cells compared with the PL and HV groups (Figure 5(d)). These results indicate that CD8^+^ lymphocytes in MM patients have a decreased ability to retain and enhance perforin following stimulation, compared with PL patients and HV.

### 3.6. Degranulation of CD8^+^ Lymphocytes

**Figure 6:**
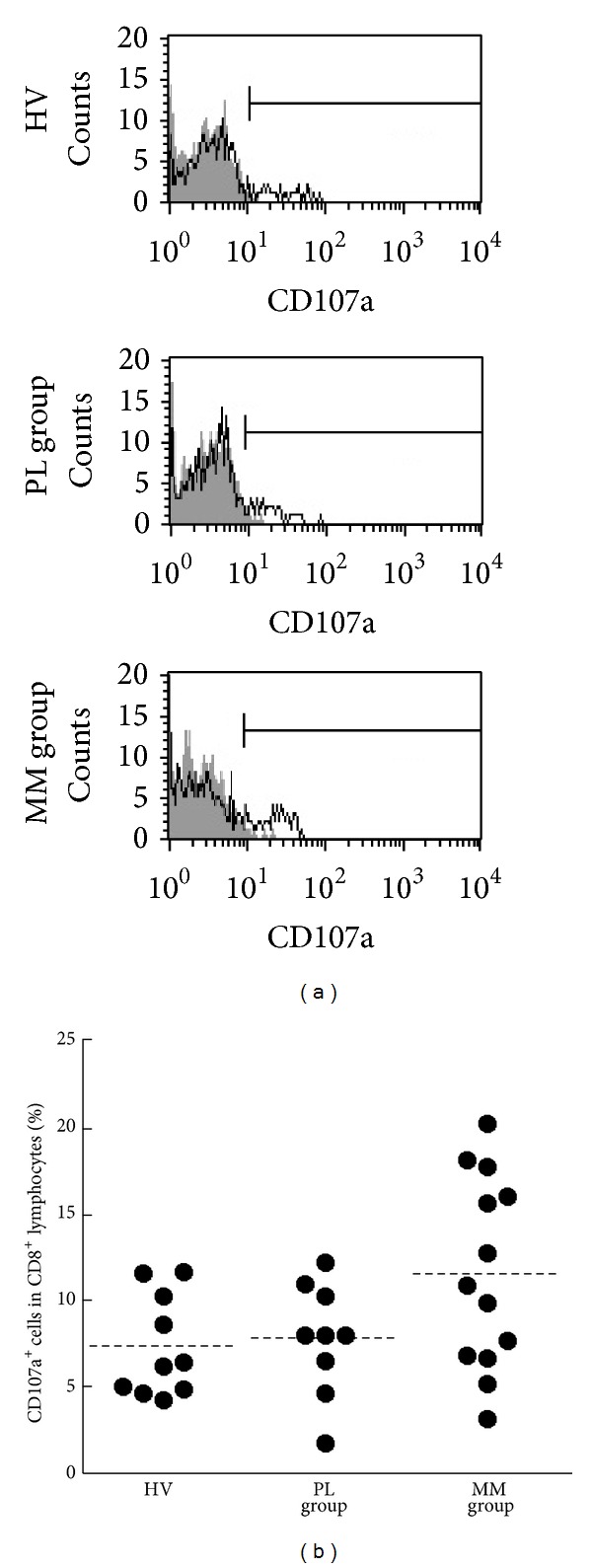
The percentages of CD107a-positive cells in CD8^+^lymphocytes of HV, PL, and MM groups. (a) Representative histograms of cell-surface CD107a in CD8^+^ lymphocytes cultured with PMA/ionomycin (solid line) and without stimulation (gray) for the HV, PL, and MM groups. (b) Representative graph showing the percentage of CD107a^+^ cells in CD8^+^ lymphocytes. Horizontal dotted bars indicate the mean percentage. Data represent values from 10 HV individuals, 9 individuals of the PL group, and 13 individuals of the MM group.

The results shown above suggested the possibility that CD8^+^ lymphocytes in MM have an insufficient ability to retain a suitable perforin level. However, we cannot exclude the possibility that the decrease in perforin in stimulated cells of MM patients might be related to augmented degranulation, by which perforin and granzymes are released outside to injure target cells. Therefore, to examine degranulation we cultured PBMCs in the presence of PMA/ionomycin stimulation for 4 h and then collected and stained cells with fluorescence-labeled anti-CD8 and anti-CD107a Abs before performing an analysis using FCM (Figure 6(a)), as described in Section 2. The percentages of CD107a^+^ cells in stimulated CD8 lymphocytes did not differ statistically in multiple comparisons among HV, PL, and MM groups (mean ± S.D., 7.4 ± 2.8, 7.8 ± 3.1, and 11.6 ± 5.4, resp.), although the percentages of CD107a^+^ cells in the MM group tended to be higher than those in PL and HV groups (Figure 6(b)). Additionally, although the MM group included some individuals who showed an enhanced decrease in perforin level after stimulation, they did not show the higher percentages of CD107a^+^ cells shown by others in the MM group or individuals of the PL and HV groups (data not shown). These findings exclude the possibility that augmented degranulation in MM patients might lead to the decrease in perforin level after stimulation.

## 4. Discussion

We previously confirmed the suppressive effect of asbestos on CD8^+^ T cells in an* in vitro* study. We therefore examined the functional properties of CD8^+^ lymphocytes in asbestos-exposed patients with PL and MM in the present study, focusing on cellularity, production of IFN-*γ*, and intracellular levels of effector molecules affecting cytotoxicity. In particular, CD8^+^ lymphocytes were also analyzed for the ability to retain/enhance the effector molecules perforin and granzyme after stimulation, as well as the storage level of those molecules in fresh cells. PL patients showed higher percentages of granzyme B^+^ and perforin^+^ cells in stimulated CD8^+^ lymphocytes, compared with HV. In contrast, the MM group showed a decrease in the percentage of perforin^+^ cells following stimulation, which differed significantly from that of the PL and HV groups. On the other hand, the PL and MM groups showed higher percentages of CD45RA^−^ and perforin^+^ cells in fresh CD8^+^ lymphocytes. These findings indicate that the characteristics of CD8^+^ lymphocytes in PL and MM patients differ, although they have several points in common. The commonality between PL and MM patients means that the increases in CD45RA^−^ and perforin^+^ cells may be related to asbestos exposure, whereas the difference in post-stimulation maintenance of perforin between these patients underlines the immunological states of PL and MM, related to the pathologies of asbestos exposed patients not or suffering from malignant mesothelioma, respectively.

The MM group showed a decrease in perforin level of CD8^+^ lymphocytes after PMA/ionomycin stimulation whereas this was not shown in PL and HV (Figure 5(d)), although the percentage of perforin^+^ cells in stimulated CD8^+^ did not differ from that of HV (Figure 5(c)). These results imply impairment in stimulation-induced persistent cytotoxicity of CD8^+^ lymphocytes in patients with MM compared to PL patients. Asbestos exposure might result in impaired function of CD8^+^ lymphocytes in patients with MM because our previous study showed the suppressive effect of asbestos exposure on* in vitro *induction of CTL. This phenomenon cannot explain why the CD8^+^ lymphocytes of patients with PL, namely, asbestos-exposed individuals without tumor, did not show functional impairment. However, as discussed below, the difference between the MM and PL groups might be explained by understanding that inhalation of asbestos might not always result in the suppressed function of CD8^+^ lymphocytes, unlike addition of asbestos into a culture of PBMCs, and that an adequate immune response might have been induced to protect tumor disease in individuals of the PL group analyzed in this study. Alternatively, the impaired function of CD8^+^ lymphocytes in MM patients might be related to immune suppression by tumor cells [25]. In fact, it has been shown that PBMCs isolated from blood samples of patients with carcinoma exhibited a significant decrease in T cell receptor-mediated cytotoxicity compared with HV [26]. Therefore, although we cannot draw a conclusion with absolute certainty, it is a notable finding that the CD8^+^ lymphocytes of MM patients, but not PL patients, showed an enhanced decrease in perforin level after stimulation, as first demonstrated by our present study.

CD45RA-negative cells in CD8^+^ T cells are defined as memory cells and can be divided into two types of cells, central memory cells and effector memory cells in peripheral blood [21, 22]. Both PL and MM groups showed higher percentages of CD45RA-negative cells in CD8^+^ lymphocytes compared with HV, and both groups also showed a higher percentage of perforin^+^ cells in these lymphocytes. Perforin expression is also known to be restricted to effector memory cells, but not central memory cells [21, 22]. Therefore, the findings of our study should be interpreted to indicate that PL and MM patients have an increased proportion of effector memory cells in peripheral blood CD8^+^ lymphocytes. It is suggested that this character of CD8^+^ lymphocytes might be related to asbestos exposure because both PL and MM patients should have been exposed to asbestos. However, the increase in effector memory CD8^+^ lymphocytes in peripheral blood may not result from the direct effect of asbestos exposure on the immune system because the results obtained by our* in vitro* study showed the suppressive effect of asbestos addition on cytotoxicity of CD8^+^ lymphocytes. It is possible that the increase in effector memory CD8^+^ lymphocytes might represent an immune response against abnormal cells and molecules caused by asbestos exposure. As mentioned above, it is noteworthy that CD8^+^ lymphocytes in MM patients showed impairment in stimulation-induced persistent cytotoxicity. Insufficient action of tumor immunity might be related to acquisition of MM following exposure to asbestos. In contrast, tumor immunity might function successfully in PL patients, who have not currently acquired MM.

Before beginning the present study, we predicted that PL patients might show the suppressed function of CD8^+^ lymphocytes because we reported previously that asbestos exposure suppressed the induction of human CTL during MLR [19]. Other researchers have observed that lymph nodes contained an amount of asbestos that was comparable to that of lung parenchyma and pleural plaques [27]. The lymph node is known as a place where naïve CD8^+^ T cells differentiate into CTL [28]. These findings support our idea that induction of CTL might suffer from exposure to asbestos* in vivo*. Contrary to our expectation, CD8^+^ T cells in PL patients showed increased percentages of granzyme B^+^ cells and maintained a high percentage of perforin^+^ cells following stimulation, which differed from HV and MM groups. As mentioned above, these results supported the possibility that tumor immunity might function well in PL patients. However, further discussion on this point is needed. In the study using MLR, CD8^+^ lymphocytes were exposed to asbestos during stimulation with allogenic targets and then examined for the effect of asbestos on cytotoxicity of these lymphocytes. On the other hand, CD8^+^ lymphocytes prepared from peripheral blood in the present study may include some lymphocytes that had been exposed to asbestos fiber in lymph nodes or lungs, and then they were stimulated* in vitro* for the analyses. This difference in timing concerning the effects of asbestos exposure on lymphocytes might have caused the variation in results between the MLR experiments and analyses of patient specimens.

## 5. Conclusion

Our present investigation is the first to show that MM patients have characteristics of impairment in stimulation-induced cytotoxicity of peripheral blood CD8^+^ lymphocytes and that both PL and MM patients have a common character of functional alteration in those lymphocytes, namely, an increase in memory cells, possibly related to exposure to asbestos. Our findings regarding the immunological properties of CD8^+^ lymphocytes related to asbestos exposure or mesothelioma might assist in the early detection of MM. The present method of diagnosis for MM is based mainly on image analysis by X-ray and CT-scan, which require training to obtain accurate results. Information about these lymphocytes can be easily and safely taken from peripheral blood. The combination of imaging and immunological analyses might carve out a new future for the diagnosis of MM.
